# Infective Endocarditis Presenting as Intracranial Hemorrhage: A Case Report

**DOI:** 10.7759/cureus.69082

**Published:** 2024-09-10

**Authors:** Divya Singh, Francesca Pastrana, Laura Polhemus, Hanan Qaqish, Wilson Rodriguez, Fajun Wang

**Affiliations:** 1 Neurology, Saint Louis University School of Medicine, Saint Louis, USA

**Keywords:** infective endocarditis, intracerebral hemorrhage, intracranial hemorrhage, mycotic aneurysm, streptococcus aureus

## Abstract

We describe a 31-year-old woman who presented with acute right-sided weakness and was found to have a subarachnoid hemorrhage in the left Sylvian fissure and an ipsilateral frontal intraparenchymal hemorrhage. CT angiography revealed an occlusion of the left middle cerebral artery’s M1 segment and a saccular aneurysm at its bifurcation. A cerebral angiogram confirmed these findings, and the patient subsequently underwent microsurgical aneurysm resection, which revealed a partially thrombosed pseudoaneurysm. Further stroke workup identified mitral valve vegetation, confirming the diagnosis of infective endocarditis.

## Introduction

Intracranial hemorrhage (ICH) is a rare but potentially fatal complication of infective endocarditis (IE), occurring in approximately 5% of all cases. Even more rarely, ICH can be the initial manifestation of IE, presenting with nonspecific symptoms or focal neurological deficits. Proposed pathophysiological mechanisms include hemorrhagic transformation (HT) of embolic infarcts, rupture of intracranial mycotic aneurysms, and septic endarteritis. Given the high mortality rates associated with this condition, early diagnosis and treatment are crucial. Establishing clear guidelines for both medical and surgical management is essential for improving short- and long-term outcomes. Our case underscores the importance of considering IE as a potential differential diagnosis in patients presenting with the combination of subarachnoid hemorrhage (SAH) and ischemic stroke.

## Case presentation

**Figure 1:**
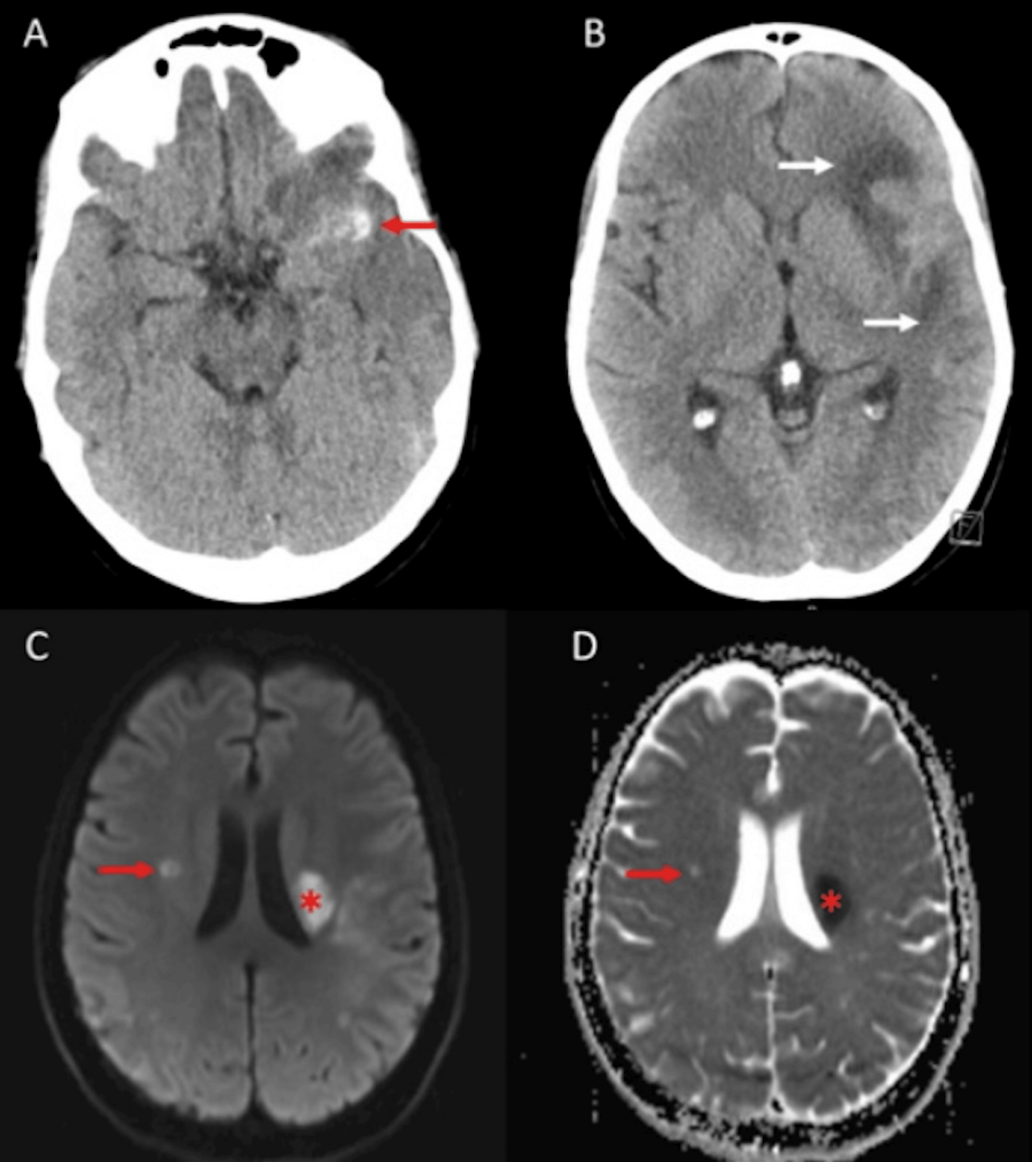
(A, B) Initial CT head without contrast showing SAH in the left Sylvian fissure (red arrow) and hypodensity in the left MCA distribution, suggesting an acute to subacute infarct (white arrows). (C, D) Brain MRI showing diffusion restriction in the left corona radiata with ADC correlation, suggestive of an acute infarct (red asterisks), and a focus of diffusion restriction in the right MCA territory without ADC correlation, indicative of a subacute to chronic infarct (red arrows). ADC: apparent diffusion coefficient; MCA: middle cerebral artery; SAH: subarachnoid hemorrhage

**Figure 2:**
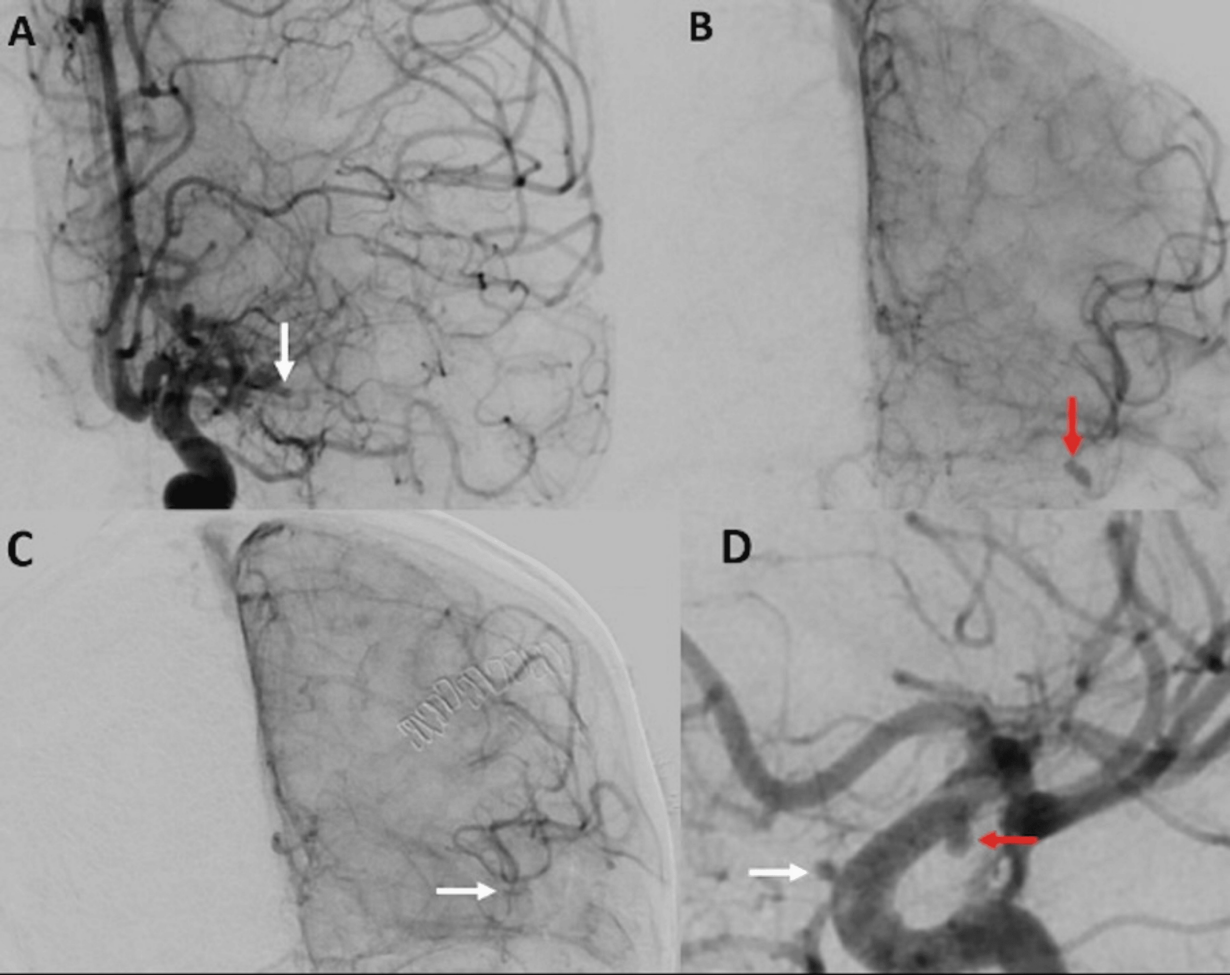
(A, B) DSA showing complete occlusion of the left MCA at the M1 segment (white arrow) and an elongated aneurysm at the left MCA bifurcation (red arrow). (C, D) DSA performed four days post-surgery showing resolution of the previously observed mycotic aneurysm at the left MCA bifurcation (C, white arrow), along with the appearance of a new mycotic aneurysm in the right ICA just above the ophthalmic segment (D, white arrow) and another at the right ICA posterior carotid wall in the supraclinoid segment (D, red arrow). DSA: digital subtraction angiography; ICA: internal carotid artery; MCA: middle cerebral artery

A 31-year-old woman with a history of elevated antiphospholipid antibodies, mitral valve prolapse, and poor dentition presented with acute onset right-sided weakness. On arrival, she was mildly tachycardic and afebrile, with a white blood cell count of 11.3 × 10^9^/L. Neurological examination revealed right upper motor neuron facial palsy and ipsilateral hemiparesis with preserved sensation. CT of the head showed a left Sylvian fissure SAH with superimposed left frontal intraparenchymal hematoma (Figure 1A) and hypodensities in the left basal ganglia and anterior temporal lobe, indicating an acute to subacute infarct (Figure 1B). CT angiography of the brain and neck demonstrated occlusion of the left middle cerebral artery (MCA) M1 segment and a saccular aneurysm measuring 5 × 3 × 3 mm at the left MCA bifurcation with retrograde filling. MRI of the brain revealed an acute infarct in the left basal ganglia and a subacute to chronic infarct in the right corona radiata (Figure 1C, 1D). SAH was noted along the bilateral frontal lobes and left Sylvian fissure. Digital subtraction angiography (DSA) confirmed occlusion of the left M1 segment and an elongated aneurysm at the left MCA bifurcation (Figure 2A, 2B).

A transthoracic echocardiogram (TTE) revealed a mobile frond-like 13 mm mitral valve vegetation, initially suspected to be nonbacterial thrombotic endocarditis. Blood cultures were collected. The patient was cleared for surgery by cardiology and successfully underwent microsurgical resection of the left MCA aneurysm, with intraoperative pathology confirming a partially thrombosed pseudoaneurysm, consistent with a mycotic aneurysm. A transesophageal echocardiogram confirmed the vegetation and revealed severe mitral regurgitation. Due to the echocardiographic findings, worsening leukocytosis, and shock, broad-spectrum antibiotics were initiated for suspected IE. Blood cultures grew *Streptococcus alactolyticus* at 31 hours, and antibiotics were de-escalated to ceftriaxone based on sensitivity results. A repeat DSA four days postoperatively demonstrated complete resolution of the clipped mycotic aneurysm at the left MCA bifurcation (Figure 2C). However, two new mycotic aneurysms were detected in the intracranial segments of the right internal carotid artery (Figure 2D). A surveillance TTE performed two weeks later showed a decrease in vegetation size. The patient was discharged to an acute rehabilitation facility and completed six weeks of intravenous ceftriaxone. A repeat cerebral angiogram five months later showed resolution of the mycotic aneurysms.

## Discussion

ICH can manifest as symptomatic primary intraparenchymal hemorrhage with or without intraventricular hemorrhage, HT, SAH, or, more rarely, subdural hematoma (Table 1). Neurological symptoms often include nonspecific presentations such as nausea, vomiting, headache, altered mentation, or more specific focal neurological deficits. Various pathophysiological mechanisms have been proposed, with the most common being HT of embolic infarcts [1-6] and rupture of intracranial mycotic aneurysms [1-9]. It is also suggested that septic endarteritis from pyogenic necrosis at the site of septic emboli may play a role, particularly during the bacteremia phase of* Staphylococcus aureus *infection, which predisposes patients to early neurological manifestations of IE [1,2]. Septic arthritis and mycotic aneurysms can be visualized on cerebral angiography, typically performed for symptomatic ICH or postmortem, suggesting that many unruptured aneurysms may go undetected and thus, their true incidence might be underestimated [1].

**Table 1:** Review of previous cases of IE complicated by ICH HT: hemorrhagic transformation; ICH: intracranial hemorrhage; IE: infective endocarditis; IPH: intraparenchymal hemorrhage; IVH: intraventricular hemorrhage; MA: mycotic aneurysm; MSSA: methicillin-sensitive* Staphylococcus aureus*; SAH: subarachnoid hemorrhage; SDH: subdural hematoma

Author	Study description and patient demographics	Epidemiology with neurologic complications	ICH on arrival	Type of ICH	ICH etiologies	Organisms identified
Hart et al. (1987) [1]	Retrospective, multicenter analysis of 17 patients with IE; 21-74 years old; 76.5% male	17 patients (100%)	7 patients (41%)	IPH (86%) and IVH (14%)	Arteritis (42%), MA (14%), and HT (29%)	*Staphylococcus aureus *(71.4%), *Streptococcus viridans *(14.3%), and *Streptococcus sanguis *(14.3%)
Salaun et al. (2018) [4]	Retrospective, single-center analysis of 963 patients with IE; 57 ± 13 years old; 75% male	225 patients (23%)	36 patients (3.7%)	Unspecified	HT (40%), MA (32%), and undetermined (28%)	*Staphylococci *(37%), *Streptococci *(38%), *Enterococci *(12%), unspecified organism (7%), and blood culture negative (6%)
Nitsch et al. (2023) [5]	Retrospective, single-center analysis of 48 patients with IE; 64.9 ± 13.94 years old; 60.4% male	48 patients (100%)	4 patients (8.3%)	IPH (75%) and SAH (25%)	HT (75%) and undetermined (25%)	*S. aureus *(45.5%) and unspecified (54.5%)
Morotti et al. (2016) [6]	Case series; three patients with IE (35-year-old male; 60-year-old male, and 44-year-old male)	-	-	SAH (33%) and IPH (66%)	MA (66%) and HT (33%)	*Enterococcus faecalis *and culture negative *S. aureus*
Flor-de-Lima et al. (2013) [7]	Case report; 17-year-old female	-	-	IPH	MA	Abiotrophia defective
Peters et al. (2006) [8]	Case report; 41-year-old male			IPH	MA	Streptococcus mitis
Kahn et al. (2011) [9]	Case report; 27-year-old male			IPH	MA	S. viridans
Yanagihara et al. (2003) [10]	Case report; 54-year-old male			SAH with SDH	Undetermined	MSSA

Several risk factors for neurological complications in IE have been described in the literature, with *S. aureus *bacteremia being the most common [1-3,5,10]. Salaun et al. reported no significant increase in the risk of ICH with *S. aureus *bacteremia, but they did observe a significant increase in the rate of ruptured mycotic aneurysms compared to *Streptococci *species [4]. Despite this finding, *S. aureus *remains the most prevalent organism associated with early neurological manifestations of IE. Coagulopathies, including anticoagulant use, antiplatelet therapy, and thrombocytopenia, are known to increase the risk of ICH and are associated with a worse prognosis [1,3-4]. Other factors correlated with an increased risk include other symptomatic systemic emboli [1,4], coexisting mitral and aortic valve involvement [2], vegetation size ≥3 cm [3], severe valve regurgitation, and intravenous substance use [4]. Notably, there is no reported increase in the risk of ICH between patients with native versus prosthetic heart valves [2]. However, patients with prosthetic or mechanical heart valves are typically on anticoagulation therapy, which consequently raises their risk of ICH.

Mortality is significantly higher in patients with neurological complications from IE compared to those without [2,5], and this risk is even greater in those presenting with ICH [3]. Additionally, ICH with an undetermined etiology carries the highest one-year mortality rate compared to unruptured mycotic aneurysms or HT [4], underscoring the importance of identifying the etiology of ICH in improving patient outcomes. Neurosurgical and cardiothoracic surgical interventions are often delayed due to the presence of ICH and the associated need to suspend anticoagulation therapy [1,3]. Currently, there are no clear guidelines on the recommended surgical intervention modalities or the timing of such procedures.

## Conclusions

To prevent delays in diagnosis, enhancing provider awareness is crucial in prompting the consideration of arteritis and mycotic aneurysm as potential causes of new SAH in patients with risk factors for IE, such as poor dentition, a history of intravenous drug use, or chronic indwelling catheters. Given the risk of significant long-term morbidity and the potential for preventable harm, it is essential to continue refining diagnostic and management strategies for ICH in patients with concurrent IE, with the goal of improving clinical outcomes.

## References

[1] Hart RG, Kagan-Hallet K, Joerns SE (1987). Mechanisms of intracranial hemorrhage in infective endocarditis. Stroke.

[2] Heiro M, Nikoskelainen J, Engblom E, Kotilainen E, Marttila R, Kotilainen P (2000). Neurologic manifestations of infective endocarditis: a 17-year experience in a teaching hospital in Finland. Arch Intern Med.

[3] García-Cabrera E, Fernández-Hidalgo N, Almirante B (2013). Neurological complications of infective endocarditis: risk factors, outcome, and impact of cardiac surgery: a multicenter observational study. Circulation.

[4] Salaun E, Touil A, Hubert S (2018). Intracranial haemorrhage in infective endocarditis. Arch Cardiovasc Dis.

[5] Nitsch L, Shirvani Samani O, Silaschi M (2023). Infective endocarditis and stroke: when does it bleed? A single center retrospective study. Neurol Res Pract.

[6] Morotti A, Gamba M, Costa P (2016). Infective Endocarditis Presenting with Intracranial Bleeding. J Emerg Med.

[7] Flor-de-Lima F, Lisboa L, Sarmento A, Almeida J, Mota T (2013). Mycotic brain aneurysm and cerebral hemorrhagic stroke: a pediatric case report. Eur J Pediatr.

[8] Peters PJ, Harrison T, Lennox JL (2006). A dangerous dilemma: management of infectious intracranial aneurysms complicating endocarditis. Lancet Infect Dis.

[9] Kahn DE, O'Phelan K, Bullock R (2011). Infectious endocarditis presenting as intracranial hemorrhage in a patient admitted for lumbar radiculopathy. Case Rep Crit Care.

[10] Yanagihara C, Wada Y, Nishimura Y (2003). Infectious endocarditis associated with subarachnoid hemorrhage, subdural hematoma and multiple brain abscesses. Intern Med.

